# Polyclonal *Burkholderia cepacia* Complex Outbreak in Peritoneal Dialysis Patients Caused by Contaminated Aqueous Chlorhexidine

**DOI:** 10.3201/eid2609.191746

**Published:** 2020-09

**Authors:** Sally C.Y. Wong, Shuk-Ching Wong, Jonathan H.K. Chen, Rosana W.S. Poon, Derek L.L. Hung, Kelvin H.Y. Chiu, Simon Y.C. So, Wing Shan Leung, Tak Mao Chan, Desmond Y.H. Yap, Vivien W.M. Chuang, Kwok-Yung Yuen, Vincent C.C. Cheng

**Affiliations:** Queen Mary Hospital, Hong Kong, China (S.C.Y. Wong, S.-C. Wong, J.H.K. Chen, R.W.S. Poon, D.L.L. Hung, K.H.Y. Chiu, S.Y.C. So, W.S. Leung, Vincent C.C. Cheng);; The University of Hong Kong, Hong Kong (T.M. Chan, D.Y.H. Yap, K.Y. Yuen);; Hospital Authority, Hong Kong (V.W.M. Chuang)

**Keywords:** Burkholderia cepacia complex, B. cenocepacia, bacteria, polyclonal, outbreak, wound infection, chlorhexidine, peritoneal dialysis, genomic sequencing, nosocomial infections, epidemiology, manufacturers, Hong Kong. China

## Abstract

Whether *Burkholderia cepacia* complex should be an objectionable organism in antiseptic solutions with acceptable total bacterial counts is controversial. By using next-generation sequencing, we documented a polyclonal *B. cepacia* complex outbreak affecting peritoneal dialysis patients in Hong Kong that was caused by contaminated chlorhexidine solutions. Epidemiologic investigations at a manufacturing site identified a semiautomated packaging machine as the probable source of contamination in some of the brands. Use of whole-genome sequencing differentiated the isolates into 3 brand-specific clonal types. Changes in exit site care recommendations, rapid recall of affected products, and tightening of regulatory control for chlorhexidine-containing skin antiseptics could prevent future similar outbreaks. Environmental opportunistic pathogens, including *B. cepacia* complex, might be included in regular surveillance as indicator organisms for monitoring environmental contamination.

*Burkholderia cepacia* is the type species of the genus *Burkholderia* and is a ubiquitous multidrug-resistant, motile, non–glucose-fermenting, gram-negative organism found in water and soil (*1*). The *B. cepacia* complex (BCC) contains >17 closely related species that require molecular methods for accurate differentiation (*2*). Previous typing methods, such as pulsed-field gel electrophoresis, restriction fragment-length polymorphism, or multilocus sequence typing (MLST), are ineffective and only enable differentiation into genome variants.

BCC is a major pathogen among patients with cystic fibrosis and an opportunistic pathogen affecting patients with indwelling medical devices and immunosuppression (*3*). Although >50 BCC-related nosocomial outbreaks associated with contaminated antiseptics or medications have been described, none of the skin disinfectant–related outbreaks were documented by next-generation genome sequencing as the typing method. The exact mode of contamination of commercial antiseptics was often not found (*4*,*5*). Implicated disinfectants and medications included intrinsically or extrinsically contaminated chlorhexidine (*4**–**11*), povidone–iodine (*12*,*13*), benzalkonium chloride (*14**–**16*), intravenous fluids or drugs (*17**–**20*), sodium docusate (*21*,*22*), eye drops (*23*), alcohol-free mouthwash, and nebulized salbutamol and albuterol (*24**–**26*).

There is a lack of consensus on whether *B. cepacia* should be considered an objectionable organism in nonsterile pharmaceutics according to guidelines for the United States and Europe (*27**–**30*). We report an outbreak involving >2 clusters of BCC strains among peritoneal dialysis patients caused by multiple brands of contaminated, prepackaged, single-use, 0.05% aqueous chlorhexidine (aqCHX) solutions.

## Materials and Methods

### Outbreak Investigation

On September 6, 2019, we conducted an investigation at the Queen Mary Hospital Dialysis Unit in Hong Kong when a cluster of 4 dialysis patients had BCC isolated from their exit site. All 4 patients had recent-onset serous to bloody discharge from their exit site (3 peritoneal dialysis catheter exit sites and 1 hemodialysis catheter exit site). The hospital is a 1,700-bed university-affiliated tertiary referral center serving ≈270 peritoneal dialysis and 110 hemodialysis patients. Noting the unusual number of BCC among renal patients, we performed case finding and established of baseline incidence rate of BCC during January 1, 2014–September 9, 2019, by using a laboratory information system (software system that records, manages, and stores data for clinical laboratories).

For the outbreak investigation, we defined a case-patient as a peritoneal dialysis patient who had BCC isolated from clinical specimens during March 13, 2018–October 30, 2019. The medical records of case-patients were reviewed by the infection control team as described (*31*). Epidemiologic investigation at the renal unit was performed, and nursing staff were interviewed and observed for any changes in their patient care practice; patients and their relatives, if available, were interviewed about their exit site care procedures. Environmental surveillance was conducted as described in the next section. Active surveillance was initiated for all peritoneal dialysis patients; we collected exit site swab specimens to screen for additional BCC cases. Ethics approval was obtained from the institutional review board of the University of Hong Kong/Hospital Authority Hong Kong West Cluster.

### Environmental Surveillance

Air, water, and environmental samples from the peritoneal dialysis unit, together with various antiseptics used for exit site care from our hospital and the community, were collected and microbiologically analyzed as described (*31**,**32*) (Appendix). In brief, we collected surface specimens by using premoistened, Polywipe sponge swabs (Medical Wire & Equipment, https://www.mwe.co.uk). We sampled faucets and drains of sinks by using transport rayon swabs (Copan Diagnostics, https://www.copanusa.com). We collected tap water (250 mL) into labeled sterile bottles. We used an air sampler, SAS Super ISO 180 model 86834 (VWR International PBI Srl, https://it.vwr.com), to collect 1,000 liters of air onto MacConkey agar (CM 0507; Oxoid, http://www.oxoid.com) containing 0.0005% crystal violet (Merck KGaA, https://www.emdgroup.com) and 4 μg/mL gentamicin (CG-MAC). We collected in-use and unopened antiseptics in the hospital. Unopened 0.05% aqCHX was also obtained from other (outside) stores.

Tap water was filtered through a 0.45-μm membrane, which was then inoculated onto CG-MAC. Sponge swabs and transport rayon swabs were incubated in sterile selective brain heart infusion broth (CM1135; Oxoid) containing 4 μg/mL gentamicin, 15 μg/mL vancomycin, and 1 μg/mL amphotericin B (G3632, V2002, and A4888, respectively; Sigma-Aldrich, https://www.sigmaaldrich.com) at 37°C overnight before inoculation onto CG-MAC. All disinfectants and antiseptics were subjected to 1:10 dilution with neutralization broth (brain-heart infusion plus 2% Tween 80 [P1754; Sigma-Aldrich], 0.3% sodium thiosulphate pentahydrate [27910.260; VWR Chemicals, https://us.vwr.com], 0.4% potassium dihydrogen phosphate [26936.260; VWR Chemicals], and 0.5% lecithin). The suspension was left at room temperature for 5 min, then 100 μL of the suspension was spread onto blood agar (CM0331; Oxoid). Water and air samples were incubated at 37°C for 1 day, followed by room temperature for 5 days. Other specimens were incubated at 37°C for 5 days and examined daily for visible bacterial growth. Any bacterial growth was further speciated, and bacterial CFUs were also counted for air and antiseptic cultures.

### Clinical Specimens

We processed all clinical specimens obtained before the outbreak investigation according to standard laboratory operating procedures. We performed active surveillance for BCC collected by swabbing catheter exit sites for all peritoneal dialysis patients. These swab specimens were inoculated onto CG-MAC for incubation at 37°C for 2 days. Patients with clinical symptoms suggestive of invasive catheter-related infection were investigated accordingly (e.g., peritoneal fluid or blood culture).

### Field Investigation at Brand B Manufacturing Site

On September 19, 2019, a joint field investigation at brand B manufacturing site was conducted by a team of field epidemiologists, infection control professionals, and clinical microbiologists. The process of reconstitution, dilution, and packaging of 5% chlorhexidine solution into individually packed 25-mL volumes of 0.05% aqCHX was directly observed. Environmental samples and antiseptics were collected for microbiological investigations as described in the previous sections.

### Identification by Matrix-Assisted Laser Desorption/Ionization Time-of-Flight Mass Spectrometry

We picked bacterial colonies from blood agar or CG-MAC for matrix-assisted laser desorption/ionization time-of-flight (MALDI-TOF) mass spectrometry identification with bacterial colony protein extraction by using a direct transfer method. We measured mass spectra of isolates by using the MBT Smart Mass Spectrometer (Bruker Daltonik, https://www.bruker.com) and the Bruker MBT Database 9.0 (8326 spectra). Scores >2.0 were considered as showing high-confidence identification and scores of 1.7–2.0 as showing low-confidence identification.

### Whole-Genome Sequencing and Bioinformatic Analysis

We further analyzed environmental and clinical BCC isolates by using the NovaSeq 6000 Sequencing System (Illumina Inc., https://www.illumina.com) at The University of Hong Kong Li Ka Shing Faculty of Medicine, Centre for PanorOmic Sciences, Genomics Core (Appendix). Two archived outbreak-unrelated BCC isolates were used as controls. We extracted MLST profiles from whole-genome assemblies by using BIGSdb, which is available on the BCC PubMLST website (*33*). We performed phylogenetic analysis according to single-nucleotide polymorphisms (SNPs) by using CSIPhylogeny version 1.4 with default settings (Appendix) (*34*). Results from CSIPhylogeny were subsequently imported into FigTree version 1.4.4 (http://tree.bio.ed.ac.uk) for visualizing the phylogenetic tree.

### Statistical Analysis

We used the exact rate ratio test to compare exit site infection (ESI) rates between centers with and without routine chlorhexidine use. A p value <0.05 was considered statistically significant. We applied the Holm-Bonferroni correction for multiple comparisons to control the familywise error rate at 0.05. We used the R package rateratio.test (https://www.r-project.org) to perform calculations. We used an independent *t*-test to compare means of outbreak durations involving nonsterile and sterile sites. We used SPSS Statistics 20 (IBM, https://www.ibm.com) to perform this analysis.

## Results

### Epidemiologic Investigation

On September 6, 2019, we launched an outbreak investigation when BCC was isolated from 3 peritoneal dialysis catheter exit sites and 1 hemodialysis catheter exit site for 4 patients (2 women and 2 men; age range 49–90 years, median age 60.5 years). The exit site swab specimens were used for investigation of suspected ESI on September 4, 2019. Three patients had BCC isolated from previous exit site specimens, 1 from as early as September 24, 2018. The number of days from catheter insertion to first isolation of BCC ranged from 300 to 2,329 days (mean 1,084.5 days, median 854.5 days).

**Figure 1:**
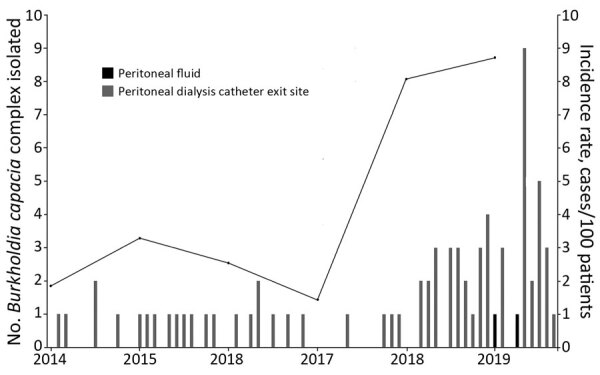
Epidemic curve and incidence rate of *Burkholderia cepacia* complex isolated from peritoneal dialysis patients, Hong Kong, China, January 2014–September 2019.

Retrospective case finding of BCC showed an increasing trend over time among nonduplicated dialysis patients since March 2018. During March 13, 2018–September 6, 2019, BCC was isolated from 53 renal patients, including 47 peritoneal dialysis catheter exit sites and 2 peritoneal fluid specimens (Table 1). The incidence rate of BCC isolated from peritoneal dialysis catheters during 2018–2019 was > 2 SD from baseline (Figure 1), confirming an outbreak of BCC among peritoneal dialysis patients. Interviews with ward staff and observation of patient care practice found no recent changes or irregularity but showed that peritoneal dialysis patients purchased 0.05% aqCHX from community stores and used this solution for routine exit site care. Brands A and B were the commonest aqCHX bought by peritoneal dialysis patients because they were the most readily available brands in the community.

**Table 1:** Specimen types and demographic characteristics for 53 renal dialysis patients from whom *Burkholderia cepacia* complex was isolated, Hong Kong, China, March 13, 2018–September 6, 2019*

Characteristic	2018, 25 patients	2019, 28 patients	Total, 53 patients
Specimen type	23 PD catheter ES; 1 HD catheter ES; 1 ES swab specimen not otherwise specified	23 PD catheter ES; 2 HD catheter ES; 2 peritoneal fluid; 1 blood culture from HD catheter	46 PD catheter ES; 3 HD catheter ES; 2 peritoneal fluid; 1 blood culture from HD catheter; 1 ES swab specimen not otherwise specified
Age, y, mean (median, range)	60.1 (65, 24–81)	65.8 (66, 46–90)	63.1 (66, 24–90)
Sex ratio, F:M	16:9	13:15	29:24
Days from PD/HD catheter insertion until first isolation of *B. cepacia* complex, mean (median, range)	1,192 (648, 58–2,349)	1,140 (769.5, 70–6,098)	1,163, (713, 58–6,198)
*B. cepacia* complex peritonitis	1	4	5
Removal of PD catheter	1	3 (2 caused by renal transplant)	4
Previous infections			
ESI caused by other organisms	8	7	15
Peritonitis caused by other organisms	4	6	10
Antimicrobial drug use <1 y before isolation of *B. cepacian* complex	19	26	45
No. deaths†	2	2	4

*ES, exit site; ESI, exit site infection; HD, hemodialysis; PD, peritoneal dialysis. †None of the 4 deaths were attributable to infection by *B. cepacia* complex.

### Environmental Surveillance

We collected 63 environmental and antiseptic specimens used in peritoneal dialysis catheter exit site care from the renal unit (Table 2). Different brands of aqCHX were purchased in the community (brands A–F) and collected in the hospital (brands G and H). All 77 aqCHX collected in the hospital were culture negative, but 103 of the 104 community aqCHX showed bacterial growth (Table 2). Brand A of aqCHX had an average bacterial load of 3.6 × 10^5^, and brand B had a value of 5.9 × 10^4^ CFU/mL. No BCC was isolated from environmental samples and other antiseptics collected from the renal unit.

**Table 2:** Environmental specimens collected and tested for investigation of *Burkholderia cepacia* complex outbreak in peritoneal dialysis unit, Hong Kong, China, March 13, 2018–September 6, 2019*

Characteristic	No. specimens	Culture result (mean, median, range), CFU/mL
Peritoneal dialysis unit		
Environment		
Air samples	2	Negative for BCC
Swab specimens from sink and faucet	12	
Water samples from sink in ward	10	
Soaps from dispensers next to patient sinks	4	
Swab specimens from wound dressing trolleys	3	
Blood pressure cuffs, gloves, and tissue paper	6 (2 each)	
Connection shield SysIIK with povidone–iodine solution†	3	
Exit site care agents		
In-use povidone–iodine	10	Negative for BCC
Single-use prepackaged saline and sterile water	10 (5 each)	
White wine vinegar	3	
Aqueous chlorhexidine		
Brand A (outside hospital)	51	43 with BCC only (3.6 × 10^3^, 1.9 × 10^2^, 2.7–7.6 × 10^4^); 4 with *Ralstonia* species only (77, 85, 46–93); 4 with BCC and *Ralstonia* species (120, 120, 94–130)
Brand B (outside hospital)	45	45 with BCC (5.9 × 10^4^, 4.6 × 10^4^, 2.9 × 10^4^–1.2 × 10^5^)
Brand C (outside hospital)	4	4 with BCC (8.3 × 10^3^, 6.8 × 10^3^, 8 × 10^2^–1.9 × 10^4^)
Brand D (outside hospital)	2	2 with BCC (2.8 × 10^5^, 2.8 × 10^5^, 2.4–3.2 × 10^5^)
Brand E (outside hospital)	1	1 with BCC (1.5 × 10^5^)
Brand F (outside hospital)	1	Negative for BCC
Brand G (from hospital)	47	Negative for BCC
Brand H (from hospital)	30	Negative for BCC
Brand B manufacturing site		
Environment		
Air samples	2	Negative for BCC
Plastic packaging	1	Negative for BCC
Plastic container in preparation room	1	Negative for BCC
Surface of fan in preparation room	1	Negative for BCC
Surface of air conditioner in preparation room	1	Negative for BCC
Specimens collected during dilution and packaging process		
5% chlorhexidine from original bottle	1	Negative for BCC
Chlorhexidine in measuring beaker	1	Negative for BCC
Distilled water	1	Negative for BCC
Diluted chlorhexidine in mixing compartment of semiautomated packaging machine ([I] in Figure 2), before mixing with stirring rod	1	BCC from enriched culture method with overnight incubation in neutralization broth
Stirring rod surface swab specimen, before mixing diluted chlorhexidine solution	1	Negative for BCC
Stirring rod surface swab specimen, after mixing diluted chlorhexidine solution	1	BCC from enriched culture method with overnight incubation in neutralization broth
Diluted chlorhexidine in mixing bowl of packaging machine, after mixing with stirring rod	1	BCC from enriched culture method with overnight incubation in neutralization broth
Newly packed 25 mL 0.05% aqueous chlorhexidine	16	16‡ with BCC 1.2 × 10^5^, 1.2 × 10^5^, 3.6 × 10^4^–2.4 × 10^5^); 3 with concurrent *Achromobacter* species

*BCC, *Burkholderia cepacia* complex. †Baxter Healthcare SA, https://www.baxter.com. ‡Three specimens had concurrent *Achromobacter* species found in culture.

### Clinical Specimens

We collected peritoneal dialysis catheter exit site swab specimens from 275 patients for BCC surveillance. A total of 62 (22.5%) patients were positive for BCC, 33.9% (21/62) of whom had a genuine infection. A total of 29.0% (18/62) were among the 53 BCC-positive peritoneal dialysis patients identified from retrospective case finding.

### Field Investigation at Brand B Manufacturing Site

**Figure 2:**
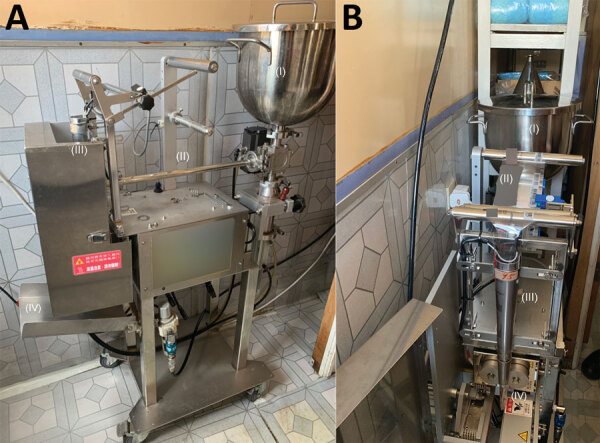
Semiautomated packing machine for aqueous chlorhexidine in brand B manufacturing site, Hong Kong, China, A) Mixing compartment (I), transfer tube from mixing compartment to dispensing end (II), area in which unused plastic packages are threaded (III), collection tray of newly packed 25 mL 0.05% aqueous chlorhexidine (IV). B) mixing compartment (I), unused plastic package (II), unused plastic package funneled to dispensing end (III), heat seal of 0.05% aqueous chlorhexidine into 25-mL packages (IV).

We observed the entire process from dilution to packaging of aqCHX. In brief, 5% aqCHX was diluted with distilled water in the mixing compartment of a semiautomated packaging machine, which channeled and packed the diluted solution into 25-mL sachets (Figure 2). Samples of antiseptics were taken before and after each step, together with additional environmental samples from the site. BCC was found in 19 of 29 environmental samples and antiseptics collected, and 3 freshly packed antiseptics also yielded *Achromobacter* species (Table 2). BCC was first detected at a low level after chlorhexidine was diluted with distilled water in the semiautomated machine, then at high level in all subsequent packaged aqCHX, implying that the machine was the probable source of contamination. No BCC was found in the distilled water, air samples, or samples taken from measuring beaker, mixing rod, and unused package material.

### Identification by MALDI-TOF Mass Spectrometry

All isolates were identified correctly to the genus level and had scores >1.7. Further species identification within the BCC was not possible.

### Whole-Genome Sequencing and Bioinformatic Analysis

A total of 80 isolates (52 patient isolates from active surveillance; 26 chlorhexidine-related isolates, including 5 isolates from the manufacturing site; and 2 outbreak-unrelated strains) were subjected to genome sequencing (Appendix Tables 1, 2). MLST analysis identified 2 predominant types. All BCC isolated from brands A, D, and E aqCHX (from the same company) were *B. cenocepacia* genomovar IIIA sequence type (ST) 1547, and all BCC isolated from brand B aqCHX and its manufacturing site were *B. cepacia* that had a novel ST (ST1693). The 2 BCC isolates from brand C were *B. cenocepacia* that had another novel sequence (ST1694).

**Figure 3:**
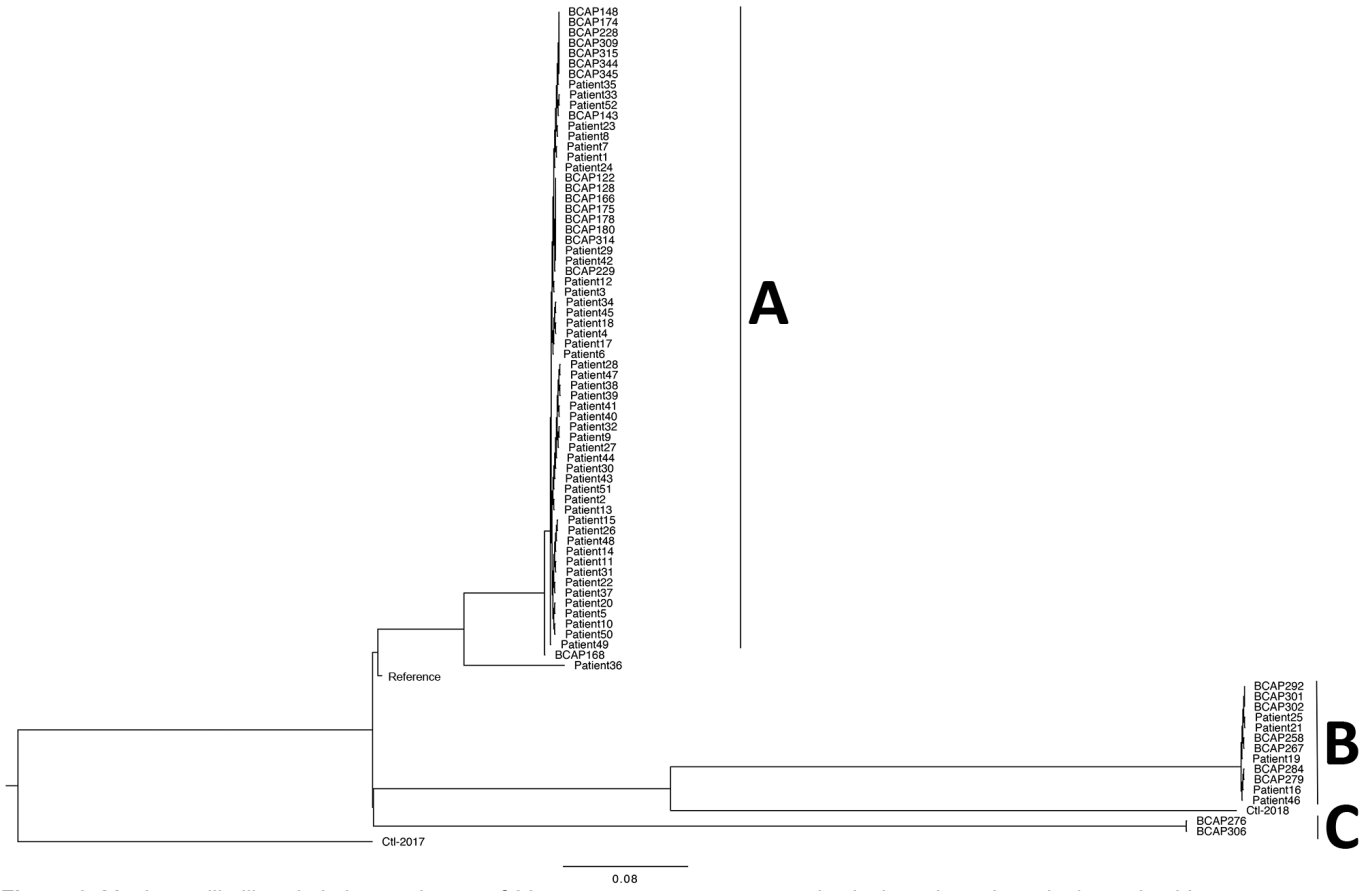
Maximum-likelihood phylogenetic tree of 80 *Burkholderia cepacia* complex isolates based on single-nucleotide polymorphisms, Hong Kong, China. A, B, and C indicate clusters. Scale bar indicates nucleotide substitutions per site.

The phylogenetic tree based on core SNPs was consistent with the MLST results showing 2 predominant clusters with highly related strains within each cluster (Figure 3). Strains from clusters A corresponded to brand A (and D and E) aqCHX and cluster B corresponded to brand B aqCHX, except that 1 brand A isolate (BCAP168) was different from cluster A strains. Both strains in cluster C corresponded to brand C aqCHX produced by a different company. A total of 47/52 patient isolates were indistinguishable or closely related to those in cluster A. Forty of these patients recalled using brand A for exit site care, 4 could not recall the brand used, and 3 reported using brand B. Of the 5 patients with isolates closely related to those in cluster B, 2 reported using brand B for exit site care, 2 reported using brand A, and 1 could not recall the brand used. The number of SNP differences in pairwise comparison of environment and patient isolates within cluster A was 0–165 and within cluster B was 0–32.

### Outbreak Control

Upon reasonable suspicion of BCC contamination affecting prepacked aqCHX purchased in the community, the renal unit called all patients to stop such a practice and arranged alternative means of exit site disinfection. On September 17, 2019, the Hospital Authority and Centre for Health Protection (CHP), Department of Health, Hong Kong, were notified of the finding of BCC in prepackaged aqCHX. Further investigation by CHP identified 183 affected patients in public and private hospitals in Hong Kong (*35*). Several additional affected brands of aqCHX were identified and voluntarily recalled by the corresponding companies (*36*). We performed snapshot ESI surveillance between centers with routine and nonroutine chlorhexidine use by using data provided by the Hospital Authority; no major difference were found between the 2 practices (Table 3). Thus, sterile saline was recommended for routine exit site care in peritoneal dialysis patients instead of aqCHX.

**Table 3:** Exit site infection rate of various microorganisms for patients in peritoneal dialysis centers in public hospitals, Hong Kong, China*

Microorganisms causing peritoneal dialysis catheter exit site infections	No. infections/1,000 patient-years		p value
	Centers with routine CHX use, n = 2,530 patients	Centers without routine CHX use, n = 2,030 patients	
Coagulase-negative staphylococci	95.25	72.41	0.0096†
Diphtheroid bacilli	19.37	31.53	0.0128
*Streptococcus* species	74.70	65.02	0.2424
Methicillin-resistant *Staphylococcus aureus*	49.41	57.64	0.2570
Methicillin-sensitive *S. aureus*	62.45	70.44	0.3241
Enterobacterales‡	95.65	86.70	0.3457
*Candida* species§	21.34	25.62	0.3990

*Values are pooled data from centers with and without routine CHX use for exit site care (deduplication done per center). CHX use included 0.05% aqueous CHX and 2% and 4% CHX body wash. CHX, chlorhexidine. †Not statistically significant after Holm-Bonferroni sequential correction (0.05/7 = 0.007). ‡Included *Citrobacter freundii*, *Enterobacter aerogenes*, *Escherichia coli*, *Klebsiella species*, *Morganella* species, *Providencia alcalifaciens*, *P. rettgeri*, *P. stuartii*, *Proteus species*, *Raoultella ornithinolytica*, *R. planticola*, and *R. terrigena*. §Included *C. holmii*, *C. valida*, *Candida* species, and *Pichia* species.

On October 8, 2019, the Guidance Notes on Classification of Products as Pharmaceutical Product under the Pharmacy and Poisons Ordinance (Cap. 138) related to chlorhexidine was revised. Skin antiseptic products containing chlorhexidine are now classified as pharmaceutical products unless otherwise stated or under certain exceptions. This guidance took effect on July 8, 2020 (*37*).

## Discussion

We report a polyclonal outbreak of BCC among peritoneal dialysis patients in our hospital that was caused by several contaminated brands of prepackaged aqCHX, which led to a territory-wide contact tracing that identified additional affected patients in other hospitals. Some observations can be made from this and previous BCC outbreaks. First, BCC outbreaks involving nonsterile sites were usually more prolonged; the mean outbreak duration was 85.4 days (median 66 days) when >50% of outbreak strains were isolated from sterile sites, compared with a mean of 245.9 days and a median of 199 days when >50% of BCC were isolated from nonsterile sites (p = 0.001) (Appendix Tables 3, 4). This finding might have occurred because BCC isolated from nonsterile sites might go unnoticed or were dismissed as sporadic, especially for patients with known risk factors, such as peritoneal dialysis catheters.

Also, the number of patients involved in an outbreak correlated with geographic distribution of the contaminated source(s). For example, 2 recent, large BCC outbreaks involving 162 and 138 patients were caused by intrinsically contaminated intravenous saline and liquid docusate (*17**,**21**,**22*); both items were distributed to multiple states in the United States. From these and previous experiences (*38*), opportunistic environmental pathogens, such as BCC and nonanthrax *Bacillus*, might be used as indicator organisms for environmental contamination and be included as part of routine surveillance.

The use of whole-genome sequencing (WGS) provided high-resolution information for further analysis of this outbreak. First, it enabled accurate identification of BCC to species level and preliminary typing of bacterial strains through MLST. Phenotypic tests and MALDI-TOF mass spectrometry are inaccurate in speciation within BCC, and unlike previous BCC outbreaks, in which identical antibiogram profiles were found among outbreak-related BCC (*4*,*39*), the antibiogram profiles among isolates from our patients were variable.

Although WGS is becoming increasingly used for outbreak investigations, the technology is not readily available in usual clinical microbiology laboratories and can be costly. Thus, alternative molecular typing methods, such as MLST or restriction fragment length polymorphism, remains the first choice for nosocomial outbreak investigations because they often provide sufficient information for evaluation of smaller scale, more focused outbreaks. In addition, these methods are also helpful for preliminary evaluation of larger outbreaks. Nevertheless, we opted for WGS in our investigation because of anticipated large-scale involvement, and the need for high-resolution data for analysis to enable rapid enforcement of corrective measures at a regional level.

Phylogenetic analysis of the WGS data based on SNP differences unambiguously differentiated the outbreak BCC isolates into distinct clusters. Combined with epidemiologic findings and field investigation at brand B manufacturing site, we believe that the contamination of aqCHX most likely occurred at their corresponding manufacturing sites. First, brand A aqCHX was manufactured outside Hong Kong and had no direct geographic linkage with the brand B manufacturing site. Second, the 5% chlorhexidine from the unopened bottle at the brand B manufacturing site did not show any growth of BCC, and presence of BCC was only detected in samples taken from the semiautomated machine, implying that the contamination had occurred during processing at the manufacturing site, rather than in the raw material. Third, the WGS analysis of clusters A and B, corresponding to brands A and B, were genetically distant. Although we cannot be certain of the exact time and duration of contamination, the retrospective case finding of *B. cepacia* isolated among our peritoneal dialysis patients during 2014–2019 showed a substantial increase only since March 2018, suggestive of a relatively recent event. We suspect that lapses in good manufacturing practices (GMPs) at various sites of chlorhexidine dilution led to bacterial contamination from the environment into the production line. BCC are ubiquitous in the environment and strains that have a MIC (>100 mg/L chlorhexidine) have been described, in which the minimum bactericidal concentration can be 3 times higher than the MIC (*40*).

The relative chlorhexidine resistance of BCC was believed to be caused by chromosomally encoded, resistant–nodulation–division efflux pumps, which up-regulate in the presence of sublethal concentrations of chlorhexidine (*41*). Thus, chlorhexidine led to the selection of a predominant BCC strain exhibiting high levels of resistance to chlorhexidine specific to each manufacturing site. In comparison, chlorhexidine has better antibacterial activities against staphylococci and Enterobacterales; thus, contamination of chlorhexidine by these organisms is rare, even at low chlorhexidine concentrations (Appendix Figure) (*42*).

The peritoneal dialysis catheter exit site care practice was revisited during this outbreak. Our local guideline stated that sterile saline and antiseptics, such as aqCHX, are acceptable (*43*), and the International Society for Peritoneal Dialysis 2017 guidelines stated that there is no evidence to suggest any antiseptics being superior in lowering the ESI rate (*44*). Some peritoneal dialysis centers have adopted routine use of chlorhexidine for ES care but a local snapshot audit on ESI rate supported the use of either sterile saline or aqCHX for exit site care.

Before the described outbreak, prepackaged aqCHX products were not considered to be pharmaceutical products in our locality because they were not labeled for use on broken skin nor had medicinal claims, and as such, these products were not registered with the Pharmacy and Poisons Board. The updated CHP guidance issued in response to this outbreak compels all chlorhexidine-containing skin antiseptic for human and animal use to be classified as pharmaceutical products unless otherwise stated, or except that these products are clearly labeled in English and Chinese for washing hands only (or equivalent); or chlorhexidine is used as a preservative or antimicrobial agent in cosmetic products, and necessitates that GMPs be observed, together with additional regulatory measures (*45*). Because terminal sterilization might inactivate or compromise the antimicrobial activity of particular antiseptics including, chlorhexidine, GMPs are relied upon to ensure the quality of the chlorhexidine produced, coupled with microbial testing of products to demonstrate their compliance with the limit laid out by the authorities (*27*,*28*,*46*). We believe that antiseptics that are potentially used on wounds, compromised mucosal surfaces, exit sites or in immunocompromised patients should be subjected to regulations as pharmaceutical products to avoid future similar outbreaks.

This study had several limitations. First, the outbreak that we described was restricted to peritoneal dialysis patients. Non–peritoneal dialysis–related infections associated with contaminated aqCHX would not have been readily identified during initial case finding. Subsequent case finding based on exposure to contaminated aqCHX identified other affected groups of patients (e.g., persons with left ventricular–assisted devices). Also, BCC isolated from peritoneal dialysis patients before September 6, 2019, and older lots of aqCHX were not available. Thus, only BCC strains identified from active patient surveillance and recent lots of prepackaged aqCHX were included for WGS. Therefore, phylogenetic analysis of the environmental and clinical strains might only reflect recent transmissions. Nevertheless, isolates subject­ed to WGS were from 12 patients who were among the 53 patients identified by the initial retrospective case finding. All of these isolates were highly related to strains within cluster A. Finally, investigation of brand C was not performed because there were no patient isolates within cluster C and none of the peritoneal dialysis patients used this brand. Other affected brands of aqCHX were imported from outside Hong Kong. Therefore, field investigation at the manufacturing sites for these brands was also not possible. Nevertheless, all affected brands were recalled and will be subject to the new regulatory measures.

In conclusion, our investigations identified a polyclonal outbreak of BCC caused by contamination of multiple brands of commercial aqCHX. The findings illustrated that genome sequencing enabled high-resolution and accurate analysis of the outbreak strains, which facilitated identification of the probable cause or point of contamination. Timely actions and coordination between renal units, the Microbiology and Infection Control Services, Hospital Authority, and Department of Health ensured prompt control of the outbreak and amendment of peritoneal dialysis catheter exit site care practice guidelines, voluntary territory-wide recall of the contaminated aqCHX, and tightening of regulatory control of chlorhexidine-containing skin antiseptics to prevent additional cases. Surveillance of environmental opportunistic pathogens, such as BCC, might enable these indicator organisms to be used to monitor environmental contamination for early detection of similar outbreaks.

Appendix Additional information on polyclonal *Burkholderia cepacia* complex outbreak in peritoneal dialysis patients caused by contaminated aqueous chlorhexidine.
