# Supplementary material for: Polyclonal Burkholderia cepacia Complex Outbreak in Peritoneal Dialysis Patients Caused by Contaminated Aqueous Chlorhexidine
Source: Emerg Infect Dis. 2020 Sep;26(9):1987–97. doi: 10.3201/eid2609.191746 (PMC7454066; doi:10.3201/eid2609.191746)
Supplement: Appendix — Additional information on polyclonal Burkholderia cepacia complex outbreak in peritoneal dialysis patients caused by contaminated aqueous chlorhexidine. [file 19-1746-Techapp-s1.pdf]

Article DOI: https://doi.org/10.3201/eid2609.191746

Polyclonal *Burkholderia cepacia* Complex Outbreak in Peritoneal Dialysis Patients Caused by Contaminated Aqueous Chlorhexidine

Appendix

Collection and Laboratory Processing of Environmental, Air, Water, and Antiseptic Samples

Environmental samples were collected by using Polywipe sponge swabs (Medical Wire & Equipment, https://www.mwe.co.uk). These swabs are sterile, premoistened, thin, flexible sponges tailor-made for sampling environmental surfaces. The sampled sponge swabs were put into sealed sterile plastic bags individually and were properly labeled before further processing in the laboratory. Samples from the faucets and drains of sinks in renal units were taken by using transport rayon swabs (Copan Diagnostics, https://www.copanusa.com) and placed in sterile selective brain heart infusion (BHI) broth (CM1135; Oxoid, http://www.oxoid.com) containing 4 μg/mL gentamicin, 15 μg/mL vancomycin, and 1 μg/mL amphotericin B (G3632, V2002, and A4888, respectively; Sigma-Aldrich, https://www.sigmaaldrich.com) (CG-BHI) before further processing in the laboratory.

An air sampler, SAS Super ISO 180 model 86834 (VWR International PBI Srl, https://it.vwr.com), was used to collect 1,000 liters of air at a rate of 180 liters of air/min for each bacterial air sampling. The air collected was directly pass onto MacConkey agar (CM 0507; Oxoid) containing 0.0005% crystal violet (Merck KGaA, https://www.emdgroup.com) and 4 μg/mL gentamicin (CG-MAC) during a 5.5-min process. Because water has been implicated in *Burkholderia cepacia*

complex (BCC) nosocomial outbreaks, 250 mL of water from sinks in renal units were collected into labeled sterile bottles for processing in the laboratory.

Both in-use and unopened antiseptics were collected from the renal unit. Unopened 0.05% aqueous chlorhexidine (aqCHX) were also collected from other units in our hospital. Because many peritoneal dialysis patients obtain their aqCHX from the community, 0.05% aqCHX was also obtained from a medical equipment store in the hospital and outside pharmacies.

Specimen Processing

The air samples on CG-MAC were incubated directly after collection at 37°C in air for 1 day and then at room temperature. Water samples were filtered by using MicroFunnel filter funnels (Pall, https://www.pall.com) through a 0.45-μm membrane. The membrane was then placed onto CG-MAC and incubated at 37°C for 1 day, and then at room temperature. All initial processing of other environmental samples was performed in class II biosafety cabinets. For each sponge swab specimen, 3 mL CG-BHI was added into a plastic bag, in which the medium was absorbed by the sponge swab specimen. The sponge swab specimen was then squeezed repeatedly for proper mixing. Then, 2 mL of suspension was extracted from the bag and incubated at 37°C overnight, then subcultured onto CG-MAC for incubation at 37°C in air. Swabs in CG-BHI broth were incubated at 37°C overnight, then subcultured onto CG-MAC for incubation at 37°C in air.

All antiseptics were processed in class II biosafety cabinets and 70% alcohol was used to disinfect the surface of the container immediately before specimen collection. Sterile needles and syringes were used to aspirate the antiseptics from the container under aseptic condition. One milliliter of the antiseptic was transferred to 9 mL neutralization broth (BHI plus 2% Tween 80) (P1754; Sigma-Aldrich), 0.3% sodium thiosulphate pentahydrate (27910.260; VWR Chemicals, https://us.vwr.com), 0.4% potassium dihydrogen phosphate (26936.260; VWR Chemicals), and 0.5% lecithin. The

suspension was left at room temperature for 5 min. Then, 100 μL of suspension was spread onto blood agar (CM0331; Oxoid) for incubation at 37°C in air.

All culture plates were incubated for ≤5 days and were examined daily for visible bacterial growth. Any bacterial growth was further speciated. For air samples and antiseptic cultures, bacterial CFUs were also counted.

Peritoneal dialysis catheter exit site swab specimens for BCC surveillance were inoculated onto CG-MAC agar upon arrival at the microbiology laboratory. The inoculated agar was incubated at 37°C in air for 2 days and examined daily for bacterial growth.

Genome Sequencing

The BCC isolates were further analyzed by genome sequencing with the NovaSeq 6000 sequencing system (Illumina Inc., https://www.illumina.com) at The University of Hong Kong. A BCC isolate from a peritoneal swab specimen from a patient with acute necrotizing pancreatitis during 2017 and a blood culture isolated during 2018 from a patient with atonic urinary bladder with recurrent urinary tract infection were included as unrelated controls.

Libraries (pair-end sequencing of 151 bp) were prepared on the basis of the protocol for the Nextera XT DNA Sample Prep Kit (Illumina). Enriched libraries were validated by using a Fragment Analyzer (https://www.agilent.com) and Qubit (https://www.thermofisher.com), and quality control analysis was performed by using a quantitative PCR. The libraries were denatured and diluted to optimal concentration. Illumina NovaSeq 6000 was used for Pair-End 151-bp sequencing.

Using software from Illumina (bcl2fastq), we assigned sequencing reads into individual samples; each sample had an average throughput of 1.7 Gb and a total throughput of 137.9 Gb. In terms of sequence quality, an average of 93% of the bases achieved a quality score of Q30, in which Q30 indicates the accuracy of a base call to be 99.9%.

Sequencing reads were filtered for adaptor sequence and low-quality sequence, followed by retaining only reads with read length ≥40 bp by using Cutadapt version 1.8.1 (*1*) and custom scripts. Low quality is defined as reads with >5% unknown bases N and reads having >50% of bases with a quality value ≤11.

De novo genome assembly was performed on samples by using preprocessed reads with SPAdes assembler version 3.13.0 (*2*). A range of k-mer sizes of 21, 33, 55, and 77 were used. The assembly yielded an average genome size of 8.1 Mb and an average N50 value of 322 Kb, and number of scaffolds ranged from 53 to 134. All assembled sequences were annotated by using Prokka version 1.14.0 (*3*) and setting genus as *Burkholderia* and species as *cepacia*. Multilocus sequence typing profiles were extracted from whole-genome assemblies by using BIGSdb (*4*), which is available on the *B. cepacia* complex PubMLST website (https://pubmlst.org/bcc/).

Phylogenetic Analysis

Scaffold sequences and reference genome sequence of BCC ST32 were uploaded to the CSIPhylogeny 0v1.4 Web site (*5*) with default settings. Results from CSIPhylogeny were subsequently imported into FigTree version 1.4.4 (http://tree.bio.ed.ac.uk) for visualizing the phylogenetic tree.

References

1. Martin M. Cutadapt removes adapter sequences from high-throughput sequencing reads. EMBnet J. 2011;17:10–12 [cited 2020 May 11]. https://journal.embnet.org/index.php/embnetjournal/article/view/200

2. Bankevich A, Nurk S, Antipov D, Gurevich AA, Dvorkin M, Kulikov AS, et al. SPAdes: a new genome assembly algorithm and its applications to single-cell sequencing. J Comput Biol. 2012;19:455–77. PubMed https://doi.org/10.1089/cmb.2012.0021

3. Seemann T. Prokka: rapid prokaryotic genome annotation. Bioinformatics. 2014;30:2068–9. PubMed https://doi.org/10.1093/bioinformatics/btu153

4. Jolley KA, Bray JE, Maiden MCJ. Open-access bacterial population genomics: BIGSdb software, the PubMLST.org website and their applications. Wellcome Open Res. 2018;3:124. PubMed https://doi.org/10.12688/wellcomeopenres.14826.1

5. Kaas RS, Leekitcharoenphon P, Aarestrup FM, Lund O. Solving the problem of comparing whole bacterial genomes across different sequencing platforms. PLoS One. 2014;9:e104984. PubMed https://doi.org/10.1371/journal.pone.0104984

6. Berkelman RL, Lewin S, Allen JR, Anderson RL, Budnick LD, Shapiro S, et al. Pseudobacteremia attributed to contamination of povidone-iodine with *Pseudomonas cepacia*. Ann Intern Med. 1981;95:32–6. PubMed https://doi.org/10.7326/0003-4819-95-1-32

7. Panlilio AL, Beck-Sague CM, Siegel JD, Anderson RL, Yetts SY, Clark NC, et al. Infections and pseudoinfections due to povidone–iodine solution contaminated with *Pseudomonas cepacia*. Clin Infect Dis. 1992;14:1078–83. PubMed https://doi.org/10.1093/clinids/14.5.1078

8. Pegues DA, Carson LA, Anderson RL, Norgard MJ, Argent TA, Jarvis WR, et al. Outbreak of *Pseudomonas cepacia* bacteremia in oncology patients. Clin Infect Dis. 1993;16:407–11. PubMed https://doi.org/10.1093/clind/16.3.407

9. van Laer F, Raes D, Vandamme P, Lammens C, Sion JP, Vrints C, et al. An outbreak of *Burkholderia cepacia* with septicemia on a cardiology ward. Infect Control Hosp Epidemiol. 1998;19:112–3. PubMed https://doi.org/10.2307/30142001

10. Kaitwatcharachai C, Silpapojakul K, Jitsurong S, Kalnauwakul S. An outbreak of *Burkholderia cepacia* bacteremia in hemodialysis patients: an epidemiologic and molecular study. Am J Kidney Dis. 2000;36:199–204. PubMed https://doi.org/10.1053/ajkd.2000.8295

11. Doit C, Loukil C, Simon AM, Ferroni A, Fontan JE, Bonacorsi S, et al. Outbreak of *Burkholderia cepacia* bacteremia in a pediatric hospital due to contamination of lipid emulsion stoppers. J Clin Microbiol. 2004;42:2227–30. PubMed https://doi.org/10.1128/JCM.42.5.2227-2230.2004

12. Ghazal SS, Al-Mudaimeegh K, Al Fakihi EM, Asery AT. Outbreak of *Burkholderia cepacia* bacteremia in immunocompetent children caused by contaminated nebulized sulbutamol in Saudi Arabia. Am J Infect Control. 2006;34:394–8. PubMed https://doi.org/10.1016/j.ajic.2006.03.003

13. Abe K, D'Angelo MT, Sunenshine R, Noble-Wang J, Cope J, Jensen B, et al. Outbreak of *Burkholderia cepacia* bloodstream infection at an outpatient hematology and oncology practice. Infect Control Hosp Epidemiol. 2007;28:1311–3. PubMed https://doi.org/10.1086/522679

14. Yang CJ, Chen TC, Liao LF, Ma L, Wang CS, Lu PL, et al. Nosocomial outbreak of two strains of *Burkholderia cepacia* caused by contaminated heparin. J Hosp Infect. 2008;69:398–400. PubMed https://doi.org/10.1016/j.jhin.2008.03.011

15. Heo ST, Kim SJ, Jeong YG, Bae IG, Jin JS, Lee JC. Hospital outbreak of *Burkholderia stabilis* bacteraemia related to contaminated chlorhexidine in haematological malignancy patients with indwelling catheters. J Hosp Infect. 2008;70:241–5. PubMed https://doi.org/10.1016/j.jhin.2008.07.019

16. Lee CS, Lee HB, Cho YG, Park JH, Lee HS. Hospital-acquired *Burkholderia cepacia* infection related to contaminated benzalkonium chloride. J Hosp Infect. 2008;68:280–2. PubMed https://doi.org/10.1016/j.jhin.2008.01.002

17. Romero-Gómez MP, Quiles-Melero MI, Peña García P, Gutiérrez Altes A, García de Miguel MA, Jiménez C, et al. Outbreak of *Burkholderia cepacia* bacteremia caused by contaminated chlorhexidine in a hemodialysis unit. Infect Control Hosp Epidemiol. 2008;29:377–8. PubMed https://doi.org/10.1086/529032

18. Sunenshine R, Schultz M, Lawrence MG, Shin S, Jensen B, Zubairi S, et al. An outbreak of postoperative gram-negative bacterial endophthalmitis associated with contaminated trypan blue ophthalmic solution. Clin Infect Dis. 2009;48:1580–3. PubMed https://doi.org/10.1086/598938

19. Martins IS, Pellegrino FL, Freitas A, Santos MS, Ferraiuoli GI, Vasques MR, et al. Case-crossover study of *Burkholderia cepacia* complex bloodstream infection associated with contaminated intravenous bromopride. Infect Control Hosp Epidemiol. 2010;31:516–21. PubMed https://doi.org/10.1086/651667

20. Souza Dias MB, Cavassin LG, Stempliuk V, Xavier LS, Lobo RD, Sampaio JL, et al. Multi-institutional outbreak of *Burkholderia cepacia* complex associated with contaminated mannitol solution prepared in compounding pharmacy. Am J Infect Control. 2013;41:1038–42. PubMed https://doi.org/10.1016/j.ajic.2013.01.033

21. Boszczowski I, do Prado GV, Dalben MF, Telles RC, Freire MP, Guimarães T, et al. Polyclonal outbreak of bloodstream infections caused by *Burkholderia cepacia* complex in hematology and bone marrow transplant outpatient units. Rev Inst Med Trop São Paulo. 2014;56:71–6. PubMed https://doi.org/10.1590/S0036-46652014000100011

22. Lalitha P, Das M, Purva PS, Karpagam R, Geetha M, Lakshmi Priya J, et al. Postoperative endophthalmitis due to *Burkholderia cepacia* complex from contaminated anaesthetic eye drops. Br J Ophthalmol. 2014;98:1498–502. PubMed https://doi.org/10.1136/bjophthalmol-2013-304129

23. Moehring RW, Lewis SS, Isaacs PJ, Schell WA, Thomann WR, Althaus MM, et al. Outbreak of bacteremia due to *Burkholderia contaminans* linked to intravenous fentanyl from an institutional compounding pharmacy. JAMA Intern Med. 2014;174:606–12. PubMed https://doi.org/10.1001/jamainternmed.2013.13768

24. Ko S, An HS, Bang JH, Park SW. An outbreak of *Burkholderia cepacia* complex pseudobacteremia associated with intrinsically contaminated commercial 0.5% chlorhexidine solution. Am J Infect Control. 2015;43:266–8. PubMed https://doi.org/10.1016/j.ajic.2014.11.010

25. Montaño-Remacha C, Márquez-Cruz MD, Hidalgo-Guzmán P, Sánchez-Porto A, Téllez-Pérez FP. An outbreak of *Burkholderia cepacia* bacteremia in a hemodialysis unit, Cadiz, 2014 [in Spanish]. Enferm Infecc Microbiol Clin. 2015;33:646–50. PubMed https://doi.org/10.1016/j.eimc.2015.02.013

26. Paul LM, Hegde A, Pai T, Shetty S, Baliga S, Shenoy S. An outbreak of *Burkholderia cepacia* bacteremia in a neonatal intensive care unit. Indian J Pediatr. 2016;83:285–8. PubMed https://doi.org/10.1007/s12098-015-1855-7

27. Mali S, Dash L, Gautam V, Shastri J, Kumar S. An outbreak of *Burkholderia cepacia* complex in the paediatric unit of a tertiary care hospital. Indian J Med Microbiol. 2017;35:216–20. PubMed

28. Song JE, Kwak YG, Um TH, Cho CR, Kim S, Park IS, et al. Outbreak of *Burkholderia cepacia* pseudobacteraemia caused by intrinsically contaminated commercial 0.5% chlorhexidine solution in neonatal intensive care units. J Hosp Infect. 2018;98:295–9. PubMed https://doi.org/10.1016/j.jhin.2017.09.012

29. Brooks RB, Mitchell PK, Miller JR, Vasquez AM, Havlicek J, Lee H, et al. Multistate outbreak of *Burkholderia cepacia* complex bloodstream infections after exposure to contaminated saline flush syringes—United States, 2016–2017. Clin Infect Dis. 2018. PubMed

30. Hamill RJ, Houston ED, Georghiou PR, Wright CE, Koza MA, Cadle RM, et al. An outbreak of *Burkholderia* (formerly *Pseudomonas*) *cepacia* respiratory tract colonization and infection associated with nebulized albuterol therapy. Ann Intern Med. 1995;122:762–6. PubMed https://doi.org/10.7326/0003-4819-122-10-199505150-00005

31. Reboli AC, Koshinski R, Arias K, Marks-Austin K, Stieritz D, Stull TL. An outbreak of *Burkholderia cepacia* lower respiratory tract infection associated with contaminated albuterol nebulization solution. Infect Control Hosp Epidemiol. 1996;17:741–3. PubMed https://doi.org/10.2307/30141547

32. Matrician L, Ange G, Burns S, Fanning WL, Kioski C, Cage GD, et al. Outbreak of nosocomial *Burkholderia cepacia* infection and colonization associated with intrinsically contaminated mouthwash. Infect Control Hosp Epidemiol. 2000;21:739–41. PubMed https://doi.org/10.1086/501719

33. Balkhy HH, Cunningham G, Francis C, Almuneef MA, Stevens G, Akkad N, et al. A National Guard outbreak of *Burkholderia cepacia* infection and colonization secondary to intrinsic contamination of albuterol nebulization solution. Am J Infect Control. 2005;33:182–8. PubMed https://doi.org/10.1016/j.ajic.2005.01.001

34. Molina-Cabrillana J, Bolaños-Rivero M, Alvarez-León EE, Martín Sánchez AM, Sánchez-Palacios M, Alvarez D, et al. Intrinsically contaminated alcohol-free mouthwash implicated in a nosocomial outbreak of *Burkholderia cepacia* colonization and infection. Infect Control Hosp Epidemiol. 2006;27:1281–2. PubMed https://doi.org/10.1086/508845

35. Estivariz CF, Bhatti LI, Pati R, Jensen B, Arduino MJ, Jernigan D, et al. An outbreak of *Burkholderia cepacia* associated with contamination of albuterol and nasal spray. Chest. 2006;130:1346–53. PubMed https://doi.org/10.1378/chest.130.5.1346

36. Kutty PK, Moody B, Gullion JS, Zervos M, Ajluni M, Washburn R, et al. Multistate outbreak of *Burkholderia cenocepacia* colonization and infection associated with the use of intrinsically contaminated alcohol-free mouthwash. Chest. 2007;132:1825–31. PubMed https://doi.org/10.1378/chest.07-1545

37. Serikawa T, Kobayashi S, Tamura T, Uchiyama M, Tsukada H, Takakuwa K, et al. Pseudo outbreak of *Burkholderia cepacia* in vaginal cultures and intervention by hospital infection control team. J Hosp Infect. 2010;75:242–3. PubMed https://doi.org/10.1016/j.jhin.2009.11.013

38. Dolan SA, Dowell E, LiPuma JJ, Valdez S, Chan K, James JF. An outbreak of *Burkholderia cepacia* complex associated with intrinsically contaminated nasal spray. Infect Control Hosp Epidemiol. 2011;32:804–10. PubMed https://doi.org/10.1086/660876

39. Lee S, Han SW, Kim G, Song DY, Lee JC, Kwon KT. An outbreak of *Burkholderia cenocepacia* associated with contaminated chlorhexidine solutions prepared in the hospital. Am J Infect Control. 2013;41:e93–6. PubMed https://doi.org/10.1016/j.ajic.2013.01.024

40. Zurita J, Mejia L, Zapata S, Trueba G, Vargas AC, Aguirre S, et al. Healthcare-associated respiratory tract infection and colonization in an intensive care unit caused by *Burkholderia cepacia* isolated in mouthwash. Int J Infect Dis. 2014;29:96–9. PubMed https://doi.org/10.1016/j.ijid.2014.07.016

41. Leong LE, Lagana D, Carter GP, Wang Q, Smith K, Stinear TP, et al. *Burkholderia lata* infections from intrinsically contaminated chlorhexidine mouthwash, Australia, 2016. Emerg Infect Dis. 2018;24:2109–11. PubMed https://doi.org/10.3201/eid2411.171929

42. Becker SL, Berger FK, Feldner SK, Karliova I, Haber M, Mellmann A, et al. Outbreak of *Burkholderia cepacia* complex infections associated with contaminated octenidine mouthwash solution, Germany, August to September 2018. Euro Surveill. 2018;23. PubMed https://doi.org/10.2807/1560-7917.ES.2018.23.42.1800540

43. Gleeson S, Mulroy E, Bryce E, Fox S, Taylor SL, Talreja H. *Burkholderia cepacia*: an outbreak in the peritoneal dialysis unit. Perit Dial Int. 2019;39:92–5. PubMed https://doi.org/10.3747/pdi.2018.00095

Appendix Table 1. Summary of library preparation for whole-genome sequencing of *Burkholderia cepacia* isolates

Characteristic	Summary
Average input DNA	1 ng
Library preparation protocol	Nextera XT DNA Library Prep Kit Reference Guide (15031942 v02)
Index system	IDT UDI Nextera Primer Pairs
Changes made to library preparation protocol	None
Sequencer model	Novaseq 6000
Run type	Pair end 151 bp

Appendix Table 2. Nature and distribution of throughput of each sample for whole-genome sequencing of *Burkholderia cepacia* isolates

Sample name	Associated brand	No. raw reads (Read1 + Read2)	Total throughput, Gb	% $\geq$Q30 bases
BCAP122	Brand A	11,950,402	1.8	94
BCAP128	Brand A	11,733,806	1.8	93
BCAP143	Brand A	6,274,224	0.9	84
BCAP148	Brand A	9,639,536	1.5	94
BCAP166	Brand A	10,257,462	1.5	93
BCAP168	Brand A	11,702,132	1.8	93
BCAP174	Brand A	9,678,166	1.5	91
BCAP177	Brand A	8,848,674	1.3	90
BCAP178	Brand A	11,773,222	1.8	94
BCAP180	Brand A	11,652,572	1.8	94
BCAP228	Brand A	11,807,954	1.8	93
BCAP229	Brand A	9,000,840	1.4	89
BCAP258	Brand B	12,118,698	1.8	94
BCAP267	Brand B	11,566,100	1.7	94
BCAP276	Brand C	12,404,438	1.9	94
BCAP279	Brand B	12,791,322	1.9	94
BCAP284	Brand B	10,421,288	1.6	93
BCAP292	Brand B	13,069,666	2.0	93
BCAP301	Brand B	9,275,042	1.4	90
BCAP302	Brand B	11,509,782	1.7	94
BCAP306	Brand C	11,978,500	1.8	93
BCAP309	Brand E	11,226,918	1.7	94
BCAP314	Brand D	13,238,160	2.0	94
BCAP315	Brand D	10,598,990	1.6	94
BCAP344	Brand A	13,624,626	2.1	94
BCAP345	Brand A	14,178,784	2.1	93
Ctl-2017	Outbreak unrelated blood culture isolate from 2017	12,130,950	1.8	94
Ctl-2018	Outbreak unrelated blood culture isolate from 2018	12,620,538	1.9	92
Patient 1	Brand A	12,806,494	1.9	94
Patient 2	Brand A	12,273,168	1.9	93
Patient 3	Patient using brand A aqCHX	10,764,710	1.6	93
Patient 4	Brand A	11,176,822	1.7	94
Patient 5	Brand A	10,950,102	1.7	92
Patient 6	Brand A	11,085,726	1.7	94
Patient 7	Brand A	11,562,168	1.7	93
Patient 8	Brand A	11,588,802	1.8	93
Patient 9	Brand A	14,410,584	2.2	94
Patient 10	Brand A	11,509,524	1.7	94
Patient 11	Brand A	5,409,852	0.8	88
Patient 12	Brand A	10,538,318	1.6	94
Patient 13	Brand B	13,177,162	2.0	94
Patient 14	Brand B	10,687,054	1.6	91
Patient 15	Brand B	12,277,634	1.9	92
Patient 16	Brand B	12,691,674	1.9	94
Patient 17	Unknown	11,599,212	1.8	94

Sample name	Associated brand	No. raw reads (Read1 + Read2)	Total throughput, Gb	% >Q30 bases
Patient 18	Brand A	11,848,136	1.8	93
Patient 19	Brand A	12,330,168	1.9	94
Patient 20	Brand A	12,214,730	1.8	93
Patient 21	Brand A and B	10,843,768	1.6	92
Patient 22	Brand A	12,736,614	1.9	93
Patient 23	Brand A	11,019,666	1.7	92
Patient 24	Brand A	11,704,104	1.8	94
Patient 25	Brand A	12,997,772	2.0	93
Patient 26	Brand A	12,828,720	1.9	94
Patient 27	Brand A	12,098,718	1.8	94
Patient 28	Brand A	9,875,416	1.5	95
Patient 29	Brand A	10,455,064	1.6	94
Patient 30	Brand A	12,178,434	1.8	94
Patient 31	Brand A	10,247,674	1.5	94
Patient 32	Brand A	11,413,460	1.7	94
Patient 33	Brand A	9,016,084	1.4	76
Patient 34	Brand A	3,923,344	0.6	86
Patient 35	Brand A	11,181,508	1.7	91
Patient 36	Brand A	8,991,288	1.4	90
Patient 37	Brand A	9,862,958	1.5	94
Patient 38	Brand A	10,110,866	1.5	92
Patient 39	Brand A	13,828,424	2.1	93
Patient 40	Brand A	12,469,812	1.9	94
Patient 41	Brand A	13,330,094	2.0	94
Patient 42	Brand A	10,407,292	1.6	94
Patient 43	Brand A	13,431,490	2.0	94
Patient 44	Brand A	12,053,012	1.8	94
Patient 45	Brand A	11,246,962	1.7	94
Patient 46	Unknown	13,231,332	2.0	94
Patient 47	Unknown	13,667,616	2.1	94
Patient 48	Unknown	12,343,144	1.9	93
Patient 49	Unknown	11,251,576	1.7	92
Patient 50	Unknown	13,368,328	2.0	94
Patient 51	Brand A	13,624,934	2.1	93
Patient 52	Brand A	9,421,366	1.4	89

Appendix Table 3. Summary of antiseptic- and medication-related *Burkholderia cepacia* complex outbreaks involving >50% sterile sites*

Year of outbreak, country	Site(s) of BCC isolation	Duration of outbreak, d†	Type of patients involved	No. affected patients	Implicated source (intrinsic or extrinsic contamination)	Multistate or multiple hospital involvement	Reference
1981, United States	Blood (pseudobacteremia)	210	Various wards	52	Povidone-iodine (intrinsic contamination)	4 hospitals	(*6*)
1992, United States	Peritoneal fluid (4) and blood (2)	25	ICU and HD center in pediatric facilities	6	Povidone–iodine (intrinsic contamination)	No	(*7*)
1993, Georgia	Blood	85	Oncology clinic	14	Multiuse IV fluid used for dilution of multiuse vial heparin flush solution (extrinsic contamination)	No	(*8*)
1998, Belgium	Blood	3	Cardiology ward	8	1 L dextrose used for heparin dilution (extrinsic contamination)	No	(*9*)
2000, Thailand	Blood (subclavicular line infection)	7	HD	9	1.5% chlorhexidine–cetrimide prepared from in pharmacy department	No	(*10*)

Year of outbreak, country	Site(s) of BCC isolation	Duration of outbreak, d†	Type of patients involved	No. affected patients	Implicated source (intrinsic or extrinsic contamination)	Multistate or multiple hospital involvement	Reference
2004, France	Blood (IV catheter as source in 75%)	210	NICU, PICU, pediatric gastroenterology	8	Contaminated condensate on the plastic stoppers in lipid emulsion	No	(*11*)
2006, Saudi Arabia	Blood	21	Tertiary care hospital	5	0.5% sulbutamol solution (intrinsic contamination)	No	(*12*)
2007, United States	Blood/intravenous catheter tips	214	Pediatric hematology and oncology practice, patients with subcutaneous port catheters	10	Multidose medications (extrinsic contamination)	No	(*13*)
2008, Taiwan	9 blood, 7 central venous catheter tips, 2 urine, 1 HD catheter tip	90	Hospital respiratory care ward and general ward	15	Extrinsic contamination of daily prepared diluted heparin solution in the ward	No	(*14*)
2008, South Korea	Blood	23	Cancer center	8	0.5% chlorhexidine solution diluted at hospital site	No	(*15*)
2008, South Korea	Blood (6), urine (1), wound (3), catheter tip (1), unknown (2)	21	Various wards, especially hemato–oncology and endocrine patients	13	Benzalkonium chloride diluted in hospital pharmacy	No	(*16*)
2008, Spain	Blood	151	HD patients	5	Contaminated deionized water used for dilution of 2.5% chlorhexidine at hospital site	No	(*17*)
2009, United States	Eye (endophthalmitis)	30	Hospital A (4)	4	Contaminated trypan blue dye from compounding pharmacy (unopened bottles were contaminated)	Yes	(*18*)
	Eye (endophthalmitis)	60	Hospital B (2)	2	Contaminated trypan blue dye from compounding pharmacy (unopened bottles were contaminated)	Yes	
2010, Brazil	Blood	88	Various wards	25	IV bromopride (antiemetics)	3 hospitals	(*19*)
2013, Brazil	Blood (4) and urine (3)	59	350-bed private tertiary care hospital	7	3% mannitol (intrinsically contaminated) for bladder irrigation	No	(*20*)
2014, Brazil	Blood	60	Hematology and BMT outpatient unit	24	Multidose vial of IV drug (extrinsic contamination) and a laminar flow cabinet	No	(*21*)
2014, India	Vitreous samples	91	Postcataract surgery patients	13	Local anesthetic eye drops	No	(*22*)

Year of outbreak, country	Site(s) of BCC isolation	Duration of outbreak, d†	Type of patients involved	No. affected patients	Implicated source (intrinsic or extrinsic contamination)	Multistate or multiple hospital involvement	Reference
2014, United States	Blood	7	350-bed private tertiary care hospital	7	Contaminated fentanyl solution (intrinsic contamination)	No	(*23*)
2015, South Korea	Blood (pseudobacteremia)	66	ICU and general wards	40	Commercial 0.5% chlorhexidine (intrinsic contamination).	No	(*24*)
2015, Spain	Blood	91	HD center	7	Chlorhexidine.	No	(*25*)
2016, India	Blood	90	Neonatal unit	12	In-use IV fluid bottles, ventilator humidifier	No	(*26*)
2017, India	Blood	240	Pediatric unit	76	Amikacin with contaminated rubber stopper.	No	(*27*)
2018, South Korea	Blood (pseudobacteremia)	42	NICU	21	Commercial 0.5% chlorhexidine (intrinsic contamination)	No	(*28*)
2019, United States	Blood	150	Skilled nursing facilities	162	IV saline (intrinsic contamination)	5 states, 59 facilities	(*29*)

*An outbreak in Lebanon was excluded because the prolonged outbreak duration was attributed to the political instability at the time of outbreak. Only reports where outbreak duration were described are included). BCC, *Burkholderia cepacia* complex; BMT, bone marrow transplant; HD, hemodialysis; ICU, intensive care unit; IV, intravenous; NICU, neonatal intensive care unit; PICU, pediatric intensive care unit.
†If exact dates are not specified in the report, the whole month will be counted toward duration of outbreak.

Appendix Table 4. Summary of antiseptic- and medication-related *Burkholderia cepacia* complex outbreaks involving ≥50% nonsterile sites*

Year of outbreak, country	Site(s) of BCC isolation	Duration of outbreak, d†	Type of patients involved	No. affected patients	Implicated source (intrinsic or extrinsic contamination)	Multistate or multiple hospital involvement	Reference
1995, United States	Respiratory tract specimen	215	Medical center	42	Nebulized albuterol (extrinsic contamination)	No	(*30*)
1996, United States	Respiratory specimens	330	Several adult ICUs in a hospital	44	Albuterol nebulization solution (extrinsically contaminated)	No	(*31*)
2000, United States	Respiratory specimens	699	Adult ICU, ventilated patients	69	Alcohol-free mouthwash (intrinsic contamination)	2 hospitals	(*32)*
2005, Saudi Arabia	Respiratory (31), blood (21), wound (2), CSF (1), eye (1), others (3) (some patients with >1 positive culture)	336	Tertiary care hospital and a 150-bed hospital	52	Albuterol nebulization solution (intrinsically contaminated)	2 hospitals	(*33*)
2006, Spain	Respiratory specimens (35), unspecified (2)	365	ICU (35) and non-ICU (2) patients	37	Alcohol-free 0.1% hexetidine mouthwash (intrinsically contaminated)	No	(*34*)
2006, United States	Respiratory tract specimen	183	Adult acute care facility (hospital A)	18	Contaminated albuterol (extrinsic contamination)	No	(*35*)
2007, United States	Respiratory specimens (83), urine (33), blood (20), tissue (3)	146	Multiple hospitals, especially ventilated patients.	116	Alcohol-free cetylpyridinium chloride mouthwash (intrinsic contamination)	22 hospitals in 9 states	(*36*)

Year of outbreak, country	Site(s) of BCC isolation	Duration of outbreak, d†	Type of patients involved	No. affected patients	Implicated source (intrinsic or extrinsic contamination)	Multistate or multiple hospital involvement	Reference
2009, Japan	Vaginal culture	61	Obstetrics and gynecology ward	17	0.025% benzalkonium chloride prepared in hospital pharmacy	No	(*37*)
2011, United States	4 Sinus and 1 tracheal aspirate	90	Pediatric hospital	5	0.05% oxymetazoline hydrochloride nasal spray (intrinsic contamination)	No	(*38*)
2013, South Korea	Sputum (10), Blood (4), CSF (1), others^ (3).	92	ICU and general wards	37	Contaminated purified water used for chlorhexidine dilution at hospital site	No	(*39*)
2014, Ecuador	Respiratory specimens	458	ICU	13	Alcohol-free chlorhexidine 0.12% mouthwash (intrinsic contamination)	No	(*40*)
2018, Australia	1 Blood and 6 respiratory specimens	61	ICU	7	Alcohol-free chlorhexidine mouthwash (intrinsic contamination)	2 hospitals	(*41*)
2018, Germany	Respiratory specimens	30	Postcardiac surgery	3	Octenidine mouthwash solution (intrinsic contamination)	No	(*42*)
2019, New Zealand	Peritoneal dialysis catheter exit sites	377	Peritoneal dialysis patients	9	4% chlorhexidine body wash (extrinsic contamination)	No	(*43*)

*An outbreak in Lebanon was excluded because the prolonged outbreak duration was attributed to the political instability at the time of outbreak. Only reports where outbreak duration were described are included). BCC, *Burkholderia cepacia* complex; CSF, cerebrospinal fluid; ICU, intensive care unit.

†If exact dates are not specified in the report, the whole month will be counted toward duration of outbreak.

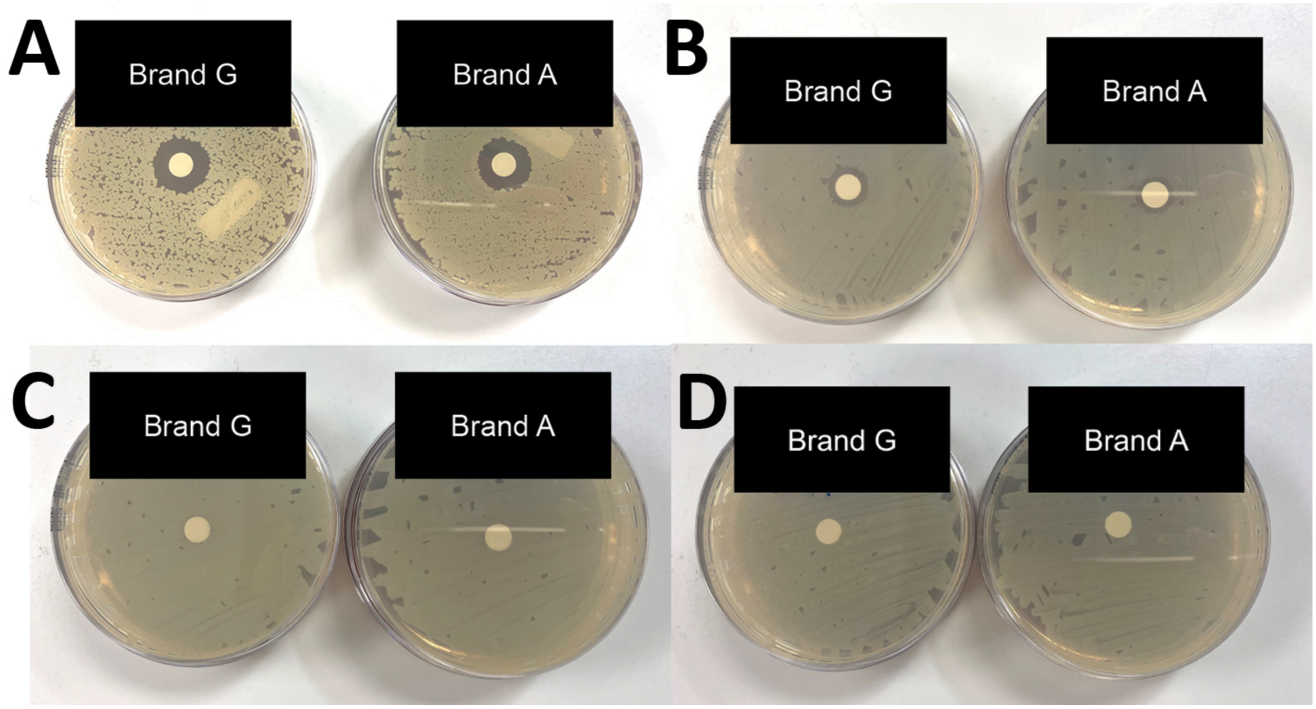

Appendix Figure. Activity of 0.05% aqueous chlorhexidine (brands G and A) against *Escherichia coli* ATCC25922, an outbreak-unrelated *Burkholderia cepacia* isolate, and an outbreak-related *B.* isolate. All plates show bacterial lawns with a 0.5 McFarland standard of the test strain against sterile filter paper disk soaked with 40 μL of aqueous chlorhexidine and incubated overnight at 37°C. A) *E. coli* ATCC25922 and large zone of inhibition. B) Outbreak-unrelated *B. cepacia* isolate and small zone of inhibition. C) Outbreak-related *B. cepacia* patient isolate, no zone of inhibition. D) Outbreak-related *B. cepacia* isolate from brand A aqueous chlorhexidine, no zone of inhibition.
